# The riddle of sphingolipid structure-function relationship in the nervous system

**DOI:** 10.1016/j.jbc.2026.113258

**Published:** 2026-06-16

**Authors:** Susana Muñoz-Gil, Stefka D. Spassieva

**Affiliations:** Department of Physiology, University of Kentucky, Lexington, Kentucky, USA

**Keywords:** sphingolipid, long-chain base, ceramide, serine palmitoyltransferase, ceramide synthase, nervous system

## Abstract

Sphingolipids are ubiquitous in the membranes of nervous system cells. They constitute about 20% of brain lipids. Perturbations of their metabolism result in dysregulation of nervous system homeostasis leading to pathologies. The structural characteristics of sphingolipid precursors, the long-chain bases, and ceramides, determine their biophysical properties and functions, as well as those of complex sphingolipids. In the current review, we are focusing on the role of chain length, saturation, and hydroxylation of sphingolipid precursors in nervous system physiology and pathology. We discuss the enzymes from the sphingolipid metabolic pathway catalyzing the reactions introducing the structural changes in the long chain bases and ceramides. We also focus on a recently discovered class of atypical neurotoxic sphingolipids as intermediates in peripheral neuropathies.

Sphingolipids are membrane components important for the structural integrity of eukaryotic cells. They are also significant bioactive molecules, signaling functions of which have been a major study area in recent decades. More than a century ago, the sphingolipid sphingosine was first described in the brain by Johann Ludwig Wilhelm Thudichum (1). To convey the riddle that this lipid poses to the human mind, Thudichum proposed the term “sphingo” as a reference to a Greek mythical creature, the Greek Sphinx (2). In the brain, sphingolipids represent about 20% of total lipids (3, 4). Therefore, it is not surprising that the names of many sphingolipids are derived from the names of nervous system structures, such as ceramides and cerebrosides from the Latin word “cerebrum” for brain, sphingomyelin from myelin, or ganglioside from ganglion. Understanding the enigma of sphingolipids in the nervous system, that started with the work of Thudichum more than a century ago, remains an ongoing scientific endeavor.

The nervous system is divided into two principal systems, the central nervous system and the peripheral nervous system (5). The central nervous system consists of the brain and the spinal cord, while the peripheral nervous system consists of peripheral ganglia and a network of nerve fibers connecting the central nervous system with the rest of the body. In broad terms, the nervous system receives, processes, and responds to sensory information and stimuli coming from the periphery and the environment (6). The main cell types of the nervous system are neurons and glial cells (7). Neurons, the principal computing units of the nervous system, are its main communicators transmitting information by electrical signals. Glial cells, on the other hand, maintain neural homeostasis, modulate neural activity, and support metabolic functions of neurons (8, 9, 10, 11). Astrocytes, microglia, ependymal cells, and oligodendrocytes are the glial cell types of the central nervous system. In the peripheral nervous system, Schwann cells, along with satellite cells, are their glial homologs. Astrocytes regulate the neuronal environment, by providing essential nutrients and removing neurotoxic substances such as excess of neurotransmitters, toxins, and cellular debris, and they are essential for maintaining the blood-brain barrier (12, 13). Microglia are the resident immune cells of the nervous system (14). Oligodendrocytes, in the central nervous system, and Schwann cells, in the peripheral nervous system, are the glial cell types forming the myelin sheath, essential for nerve conduction along the axons (15, 16). The ependymal cells provide nutrients and growth factors and are important for the circulation and homeostasis of the cerebrospinal fluid (17). Finally, the satellite cells surround and support the neurons in the peripheral nervous system regulating their chemical environment (18, 19). Neurons and glial cells are in constant interaction. The proper neuron-glial communication is critical for the homeostasis of the nervous system and if perturbed can lead to neurological disorders (20).

In the nervous system, sphingolipids are critical for brain development, cell differentiation, cell-cell recognition and adhesion, cellular growth and survival, synaptic transmission, myelination, and apoptosis (21, 22, 23, 24, 25). In addition, minor classes of atypical sphingolipids described in the last decade, the 1-deoxysphingolipids and 1-deoxymethylsphingolipids, are identified as neurotoxic intermediates in peripheral neuropathies (26, 27, 28, 29). Individual sphingolipids differ in their structure, biophysical properties, and biological roles (30, 31, 32). The long-chain bases (LCBs), also called sphingoid bases, and ceramides serve as precursor metabolites for complex sphingolipids or can be phosphorylated to generate signaling molecules such as sphingosine-1-phosphate (S1P) and ceramide-1-phosphate (C1P) (30, 31, 33). In the current review, we will explore the significance of LCBs and ceramides structural diversity, composition, and ratios for the nervous system homeostasis (21, 22, 34, 35). More recent research started to elucidate the importance of LCBs and ceramides heterogeneity for the function and identity of individual nervous system cell types (36, 37, 38, 39). The metabolism of sphingolipids is a complex web of interdependent nodes, such as specialized enzymes and distinct sphingolipid metabolites, which play a key role in homeostatic function of the cell, including the cells of the nervous system. A disruption in a single node of the sphingolipid metabolic web can have critical consequences for cells' homeostasis and can result in neurological diseases such as Niemann–Pick, Gaucher, Sandhoff, Parkinson’s, Alzheimer’s, Huntington diseases, multiple sclerosis, and ALS, or peripheral neuropathies (21, 40, 41, 42). Complex sphingolipid metabolism and its related pathologies, such as sphingolipidoses, are historically well studied. This review will focus on a lesser explored area of sphingolipid research: how the metabolism and structure of the sphingolipid precursor metabolites, the LCBs and ceramides, affect their role in physiology and pathology in the nervous system. We will highlight the recent advances and will put into focus the outstanding questions in this field of sphingolipid biology.

## Sphingolipid metabolism

**Figure 1:**
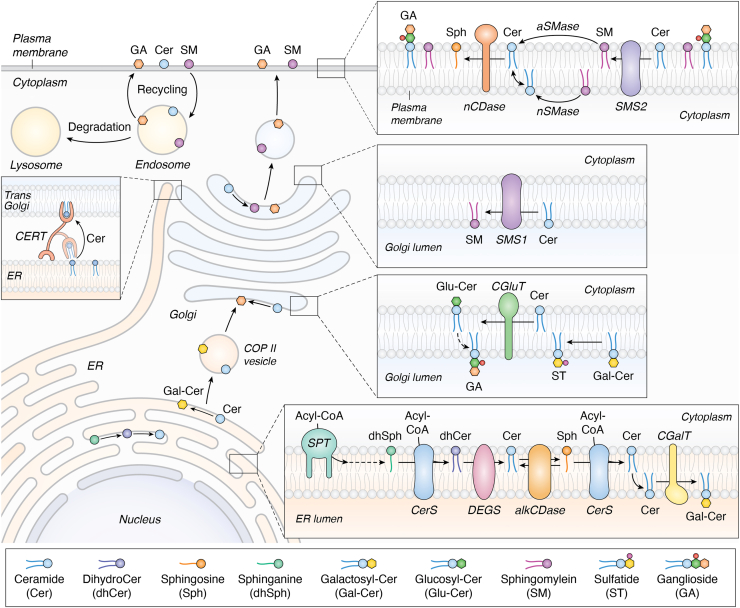
**Sphingolipid metabolism.** alkCDase, alkaline ceramidase; aSMase, acid sphingomyelinase; CerS, ceramide synthase; CGalT, UDP-galactose: ceramide galactosyltransferase; CGulT, UDP-glucose: ceramide glucosyltransferase; DEGS, dihydroceramide desaturase; nCDase, neutral ceramidase; nSMase, neutral sphingomyelinase; SMS1, sphingomyelin synthase 1; SMS2, sphingomyelin synthase 2; SPT, serine palmitoyltransferase.

The first enzyme of the *de novo* sphingolipid pathway is serine palmitoyl transferase (SPT) (Fig. 1) (43). The mammalian SPT complex is composed of two major subunits, SPT long chain base subunit 1 (SPTLC1) and either SPT long chain base subunit 2 (SPTLC2) or SPT long chain base subunit 3 (SPTLC3) and one small subunit, either SPT small subunit a (SPTssa) or SPT small subunit b (SPTssb) (44, 45, 46, 47). SPTLC1 and SPTLC2 are ubiquitously expressed, including in the nervous system, while subunit SPTLC3 is tissue specific, for example, in the skin and in the placenta (48). SPT utilizes two types of substrates, an amino acid, for example, L-serine, and a fatty acyl (FA)CoA (43, 49). The subunit composition of the SPT complex determines the FACoA substrate specificity. The small subunit SPTssa in complex with SPTLC1 and SPTLC2 has higher preference for C_16_ FACoA substrate generating 3-ketosphinganine. In the subsequent reaction catalyzed by 3-dehydrosphinganine reductase 3-ketosphinganine is reduced to the C_18_ amino alcohol sphinganine (dihydrosphingosine). As a result, mammalian sphingolipids predominantly contain C_18_ LCBs (33, 50, 51). The SPT complex with the SPTssb can utilize C_18_ FACoA in addition to C_16_ FACoA, which leads to generation of C_20_ LCBs (44, 52). The SPTLC3 subunit provides specificity to a shorter chain-length FACoA, like C_14_ myristoyl-CoA, which results in C_16_ LCBs and the respective sphingolipids (53, 54). SPTLC3 subunit has also been linked to the formation of LCBs with 12 or 20 carbon atoms, which were found in the tissues where SPTLC3 is expressed (48, 50, 55, 56). Homeostatic regulation of SPT depends on the ORMDL proteins (57, 58, 59, 60). ORMDLs sense elevated sphingolipid metabolites and regulates the SPT by inhibiting its activity (61, 62).

In a subsequent step of *de novo* sphingolipid synthesis, sphinganine is N-acylated by the ceramide synthases (CerSs) enzyme family, generating dihydroceramides (Fig. 1) (51). In mammals, there are six CerS isoforms, each with a specific affinity for the chain-length of their FACoA substrate, generating dihydroceramides with a FA moiety from C_14_ to C_26_ (Table 1). In the next metabolic step, a 4,5-trans-double bond is introduced into the sphinganine moiety of dihydroceramides by the dihydroceramide desaturase (DEGS) enzyme, leading to the production of ceramides (Fig. 1) (63, 64, 65). CerS and the ceramide content are differentially distributed in tissues and cells (66). Interestingly, the levels of ceramides with distinct FA chain-lengths do not always correlate with the levels of CerS expression that generates them, suggesting that there are other factors besides CerS expression, determining the levels of the FA chain-length of ceramides and sphingolipids in a specific tissue or organ. For example, CerS2 expression in the brain, testis, and skeletal muscle is relatively low, but the level of its downstream products, C_22_ and C_24_ hexosylceramides is unexpectedly high (67). A negative correlation was found between protein levels of CerS6 and the amount of C_16_ sphingolipids and CerS2 and C_24_, sphingolipids (68). The mechanisms of these discrepancies are not exactly known; however, posttranslational modification of CerS, regulation between different CerS isoforms or other compensatory mechanisms might be involved. One of the proposed mechanisms suggests regulation of CerS activity through formation of dimers between two different CerS isoforms (69). In another example, S1P was shown to inhibit CerS2 activity (67). CerS isoforms can increase their expression levels as a compensatory mechanism to offset the downregulation of another CerS. For example, downregulation of CerS2 leads to increased expression of CerS4, 5, and 6 (70). The compensatory mechanism aims at restoring the total dihydroceramide or ceramide levels, but with a changed FA chain-length composition.

**Table 1:** Fatty acyl chain-length specificity of mammalian ceramide synthases

Ceramide synthase isoform	FA chain-length of dihydroceramide/ceramide products	References
CerS1	C_18_	(314, 315, 316)
CerS2	C_22_, C_24_, and C_26_	(67, 70, 234, 317)
CerS3	C_24_	(318, 319, 320)
CerS4	C_20_, C_22_	(67, 70, 315, 317)
CerS5	C_14_ to C_18_	(317, 321, 322)
CerS6	C_14_, C_16_	(315, 317)

Sphingolipid metabolism is a compartmentalized process (Fig. 1). While sphinganine is produced through the *de novo* pathway located in the endoplasmic reticulum, sphingosine is a sphingolipid degradation product generated by ceramidase-mediated hydrolysis of ceramide (71, 72, 73). There are three types of ceramidase isoforms: acid, neutral, and alkaline. The localization of the specific types of ceramidases depends on their pH dependence (74, 75, 76). As a result, sphingosine can be produced by acid ceramidase in acidic compartments, such as late endosomes and lysosomes (77). The neutral ceramidases generate sphingosine at the plasma membrane (78). The alkaline ceramidases act in the endoplasmic reticulum and the Golgi (79). Ceramidases can also form ceramides from sphingosine since the ceramidase-mediated catalysis is a hydrolase reaction and as such is reversible (80, 81). The direction of the reaction depends on substrate or product availability, therefore, if sphingosine is available in excess, ceramidase reaction results in the production of ceramide. In addition to the *de novo* pathway, as mentioned above (82, 83), ceramides can be generated by the salvage pathway, which recycles sphingosine generated through the degradation of complex sphingolipids (84, 85). In the salvage pathway, CerSs utilize sphingosine as a substrate to generate directly ceramides (Fig. 1). Finally, ceramides can be generated by the degradation of complex sphingolipids like sphingomyelins, glycosphingolipids, and sulfatides (84, 86).

**Figure 2:**
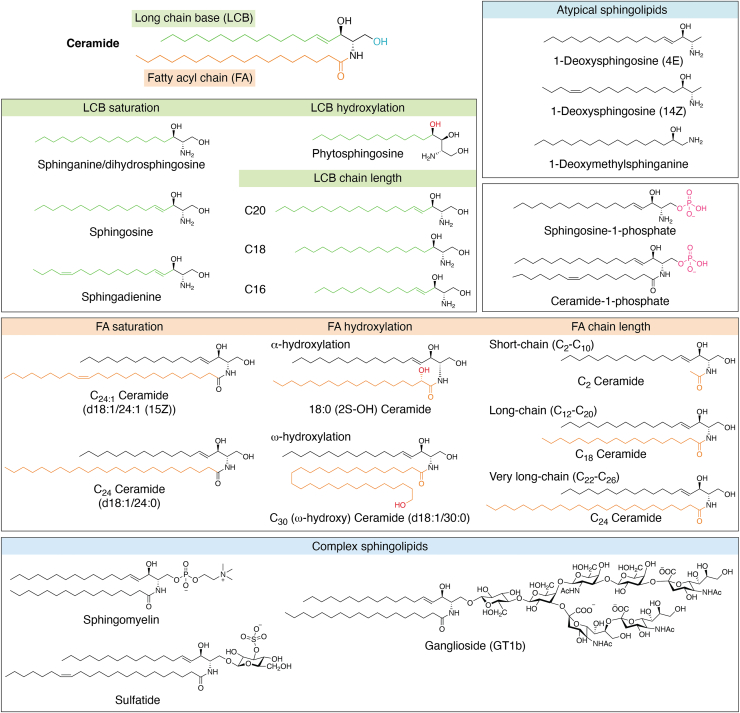
**Chemical structures of sphingolipids discussed in the review**.

The LCBs, sphinganine (dihydrosphingosine) and sphingosine, can be phosphorylated at their C_1_ hydroxyl group by sphingosine kinases resulting in dihydrosphingosine-1-phosphate (dhS1P) and S1P, respectively (Fig. 2) (55, 87). The exit from the sphingolipid metabolic pathway is catalyzed by S1P lyase, which converts S1P to hexadecenal and ethanolamine phosphate (87, 88). S1P is a bioactive molecule involved in several signaling processes (89). For instance, in the nervous system, S1P has been shown to regulate important cellular functions, that is, cell survival, blood-brain barrier maintenance, regulation of neurotransmitters release, modulation of neuronal autophagy, nociceptive responses, neurogenesis, and neuroprotection (90, 91, 92, 93). In recent years, the S1P signaling pathways mediated by its receptors, the S1P receptors (S1PRs), and by internal signaling have been extensively studied for its potential to provide therapies for diseases such as multiple sclerosis and neuroinflammation (91, 94). There are five S1PR subtypes. All five S1PRs are present in the nervous system; however, their expression varies among the nervous system cell types mediating different responses (95, 96). For example, S1PR_1_ is mostly expressed in astrocytes, where it has been shown to modulate neuroinflammatory responses in spinal cord injury models and in neuropathies (97). Neural progenitors express S1PR_1_ and S1PR_2_, while differentiated neurons express S1PR_1_ and S1PR_3_ (98). S1PR_3_ is also expressed in astrocytes, while S1PR_4_ and S1PR_5_ expressions in the brain are lower than the other three S1PRs (99, 100). S1P signaling through its receptors has been involved in regulation of growth cone formation, neurite extension, and retraction (101). S1P signaling has been implicated in neuropathic pain and neurodegenerative diseases (102, 103, 104, 105). The S1PRs have been suggested as possible targets to protect neurons and glial cells from pathological effects. For example, S1PR_1_ inhibition partially protects against paclitaxel-induced neurite damage in iPSC-derived human sensory neurons (106). In astrocytes, S1PR_1_ inhibition reduces pro-inflammatory cytokines, leading to reduced neuropathic pain (106, 107, 108, 109). S1PR_2_ has been investigated for its role in the control of spontaneous synaptic activity, in disruption of cerebrovascular integrity, and in attenuation of chemotherapy neuropathy (100, 110, 111).

Ceramides can be phosphorylated by ceramide kinase at the C_1_ position to form C1P (Fig. 2) (112). Similarly to S1P, C1P has signaling function in cell growth and survival, but compared to S1P it is understudied (112, 113). In general, C1P is considered a potent inhibitor of apoptosis, and it has been implicated in the control of macrophages and inflammatory responses (112, 114). In the nervous system, C1P and its related enzymes ceramide kinase and ceramide phosphatase, were found in synaptic vesicles (115, 116). In addition, earlier research has shown that C1P is produced in cerebellar granule cells (117).

Ceramides, after being generated in the endoplasmic reticulum, are transported to the Golgi to be further modified to complex sphingolipids (Fig. 1). The endoplasmic reticulum to the Golgi transport of ceramides is *via* two ways, by COPII vesicular transport and by ceramide transfer protein (CERT) (118). CERT-mediated ceramide transport is associated with sphingomyelin generation, while ceramide transferred by COPII vesicles is primarily converted to glycosphingolipids, such as gangliosides (119, 120). After complex sphingolipids are generated in the Golgi, they are transported to the plasma membrane by membrane-bound transport vesicles (118, 119, 121). Galactosylceramides, on the other hand are generated in the endoplasmic reticulum and subsequently converted to sulfatides in the Golgi (122, 123).

Ceramide is converted into sphingomyelin by the addition of the phosphocholine headgroup to the C_1_ OH group of ceramide (Fig. 2) (124, 125). This process can take place in the Golgi and is catalyzed by the enzyme sphingomyelin synthase 1, or at the plasma membrane by sphingomyelin synthase 2 (Fig. 1) (126). Sphingomyelin is the most abundant plasma membrane sphingolipid. It is important for myelin integrity and functional maintenance, and it has been proposed as myelin biomarker in the cerebrospinal fluid to assess demyelinating neuropathies (127). It is also involved in axonal maturation (128, 129). Sphingomyelin from breast milk has been studied for its possible role in brain development (129, 130, 131). High levels of sphingomyelin and ceramides were found in spinal cord tissue of patients with ALS and in an ALS mouse model, and were shown to increase oxidative stress and to induce motor neurons cell death (132).

Glycosphingolipids are another major group of complex sphingolipid, where sugars are attached to the C_1_ OH group of ceramide (Fig. 2) (133). Glycosphingolipids are involved in regulating apoptosis, cell proliferation, endocytosis, intracellular transport, and inflammation (130, 133). Gangliosides, glycosphingolipids which sugar head groups containing sialic acid, are essential for the nervous system and play a role in brain development (129, 134). Gangliosides have been shown to regulate neuroprotective processes, neurite outgrowth, cognitive sustainability, modulating neurotransmitter dynamics, and calcium homeostasis (135, 136). In addition, α-synuclein and amyloid beta can bind to gangliosides (137). Dysregulation of ganglioside metabolism has been associated with impaired neuropsychological behavior, axonal degeneration, myelination defects, motor deficits, and neuropathy (133, 134). Mutations in genes involved in ganglioside metabolism have been associated with diseases like infantile-onset symptomatic epilepsy syndrome, neurological deterioration, motor and cognitive impairment, hereditary spastic paraplegia, cortical atrophy, Parkinson’s, Alzheimer’s diseases, multiple sclerosis and ALS, Huntington’s disease, depression, anxiety, and stroke (135, 138).

Another class of glycosphingolipids are galactosylceramides (galactocerebrosides), which contain galactose in its head group and are synthesized in the endoplasmic reticulum by the enzyme UDP-galactose:ceramide galactosyltransferase (Fig. 1) (123). Galactosylceramides are produced by oligodendrocytes and Schwann cells and are an important lipid class in the myelin sheath (139). Galactosylceramides and their sulfatide derivatives contribute to myelin formation and stability (140, 141). Galactosylceramides are considered developmental markers because during brain development their levels are proportional to the amount of myelin (139, 142, 143). Moreover, galactosylceramide-enriched domains play a role in regulating oligodendrocyte differentiation (144, 145). Considering their central role in myelination, galactosylceramides have been studied in the context of motor learning. Galactosylceramides increase at the middle and late stages of motor learning, in contrast to another complex sphingolipid, sphingomyelin, which is increased at the early stages of the process (146, 147). Interestingly, galactosylceramides were depleted in the satellite cells of a rat diabetic neuropathy model and the authors suggested that depletion contributed to impaired neuron–glial interaction (148).

Complex sphingolipids are primarily degraded in lysosomes (Fig. 1), where they undergo hydrolysis of their head groups by specific enzymes (88). For example, sphingomyelinases hydrolyze the phosphocholine head group of sphingomyelins, while specific glycosidase enzymes, such as β-glucosidases and galactosidases, remove sugar residues from glycosphingolipids (33, 149, 150, 151). Dysregulation of galactosylceramides and sulfatides lysosomal degradation can result in pathologies, such as Krabbe disease (152, 153). Krabbe disease is a globoid cell leukodystrophy, which is characterized by mutations in lysosomal galactosylceramidase gene (*GALC*). In Krabbe disease pathology, lysosomal galactosylceramidase activity is decreased resulting in accumulation of neurotoxic psychosine, a lyso galactolipid, leading to demyelination and white matter degeneration (152, 153, 154, 155, 156). Mice lacking *Galc* gene show neurodegeneration, neuroinflammation, psychosine accumulation, and myelin abnormalities (157). In general, disruption of the sphingolipid degradation process can lead to neurodegeneration and sphingolipid lysosomal storage disorders, like Niemann–Pick type C disease, Gaucher disease, and Sandhoff disease (88, 158, 159, 160).

## LCBs in the nervous system

LCBs with a specific chain-length differ in their tissue distribution (44, 48). C_18_ LCBs, such as sphinganine and sphingosine, are the most common, including in the brain (50). In addition to C_18_ LCBs, a low abundance C_20_ LCBs (Fig. 2) have been found in brain gangliosides (161, 162). Gangliosides with C_20_ LCB are distributed along the dentate gyrus and the entorhinal-hippocampus projections (161, 163, 164). The C_20_/C_18_ LCB ratio in gangliosides influences membrane composition and, importantly, plays a role in neurodevelopment (162, 164). For instance, in rat brains, it was shown that during early development, the C_20_/C_18_ LCB ratio in gangliosides was low, but throughout the lifespan it showed an age-dependent increase (161, 164). Moreover, the glycosphingolipid expression patterns change in brains during embryonic and postnatal stages (134, 164). Measurements with matrix-assisted laser desorption/ionization imaging mass spectrometry in murine hippocampus showed that ganglioside species containing C_20_ LCBs accumulate with age (161, 163, 164). Based on these data, accumulation of gangliosides with C_20_ LCBs have been proposed as a marker for brain aging (164). Moreover, alterations of ganglioside levels have been found to be associated with Alzheimer’s disease and stroke (165, 166). For more information on the potential of gangliosides and glycosphingolipids in general and as therapeutic targets for neurodegenerative diseases, we suggest the following comprehensive reviews (135, 167, 168).

Overexpression of the small SPT subunit, SPTssb results in increased levels of C_20_ LCBs (48). In a different study, a mutation in the *Sptssb* was shown to result in elevated levels of C_20_ LCBs in ceramides and in complex sphingolipids, including glycosphingolipids and sphingomyelin, leading to neurodegeneration in the brain and retina (169). The neurodegenerative effects due to an excess of C_20_ LCB were manifested as an abnormal accumulation of neurofilaments in cerebellar white matter, suggesting axonal degeneration. In addition, in the neurons in several regions of the brain, for example, brainstem, midbrain, and thalamus, *Sptssb* mutation and elevated levels of C_20_ LCB resulted in abnormal membrane structures and vacuolization.

Disruption of SPT activity and excess sphingolipid production has been associated with juvenile ALS (57). Juvenile ALS is an early-onset motor neuron disorder, which is characterized by upper and lower motor neuron degeneration, causing muscle weakness and movement difficulties (170). The cause for juvenile ALS is not entirely known; however, several *SPTLC1* and *SPTLC2* variants are implicated (57, 58, 171, 172, 173, 174). For example, some of the *SPTLC* variants that have been associated with juvenile ALS are *SPTLC1* c.58G > A, p.Ala20Thr, p.Leu38Arg, p.A20S, p.Y23F, p.L39del, p.S331Y, and *SPTLC2* c.778G > A, p.Glu260Lys, p.G435V, c.197T>G, p.T66R, c.778G>A, p.E260K (57, 173, 174, 175, 176, 177). In addition to sphingolipids, such as sphingosine, ceramide, sphingomyelin, galactocerebrosides, and gangliosides, cholesterol also accumulates in the spinal cord of postmortem ALS patient and in animal models of ALS (132, 178, 179).

We developed a transgenic mouse model which effectively separated the effects of CerSs substrates, the LCBs, from their products, ceramides (180). This model showed that when LCBs and their phosphate derivatives (S1P and dhyhydroS1P) are elevated several folds above brain physiological levels, they can cause a neurodegenerative ataxia phenotype with progressive Purkinje neurons loss. In the rest of the neurons, the elevated LCBs and their phosphate derivatives lead to pan-neuronal membrane abnormalities and accumulation of lipofuscin, an aging and senescence marker (181, 182). These data suggest that different neuronal types have different sensitivity to elevated LCBs or alternatively, different capacity for a compensatory survival mechanism that needs to be explored further in future research.

## Ceramides in the nervous system

Ceramides play important roles in cellular processes, not only for being the main building blocks of complex sphingolipids, but also as signaling molecules, taking part in biological processes such as neural cell polarity, survival, or death (24, 183, 184, 185). Importantly, changes in ceramide levels or disruption of its metabolism have been associated with neurological pathologies.

CerS1, which catalyzes the generation of C_18_ ceramide, is expressed in neurons and as a result, the most abundant ceramide in the brain is C_18_ ceramide (Fig. 2) (38, 82, 186, 187, 188). Of note, CerS1 is also expressed in the skeletal muscle and in testis (82, 186). In mice, a catalytically inactive CerS1 results in pan-neuronal membrane abnormalities, ubiquitin and lipofuscin deposits leading to neurodegeneration, cerebellar ataxia, and in humans causes a rare genetic neurodegenerative disease, progressive myoclonic epilepsy type 8 (ORPHA #424027; OMIM #616230) (181, 189, 190, 191, 192). In the nervous system, CerS1, CerS4 and CerS6 have low expression levels in the white matter, unlike CerS2, which is localized in the white matter and predominantly expressed during myelination in oligodendrocytes and Schwann cells (187). CerS2 generates very long-chain C_22-24_ ceramides (Fig. 2). CerS5, which generates C_16_ and C_18_ ceramides, is expressed in all brain areas, including both gray and white matter, but unlike CerS2, it is not associated with active myelin synthesis. CerS6, shown to generate C_14_ and C_16_ ceramides, is expressed in the hippocampus and in the cerebellum in Purkinje neurons (66, 193). Out of the six CerS isoforms, the brain does not have detectable levels of CerS3. Instead, CerS3 is primarily expressed in peripheral tissues such as skin and testes (64, 82). This review focuses on ceramide metabolism and CerSs in the nervous system. For general information on CerSs tissue distribution, function, regulation, and disease implications, we suggest the following excellent reviews (64, 82).

A recent study in iPSC-derived neurons and glial cells measured their CerSs expression levels and ceramide profiles (35). The study found that the ceramide profile of iPSC-derived neurons differed from that of iPSC-derived glial cells. In iPSC-derived neurons, the study showed that C_16_ and C_18_ are the predominant ceramides, while iPSC-derived-astrocytes contained high levels of C_24_ ceramide and iPSC-derived-microglia C_24:1_ ceramide (Fig. 2). In addition, the iPSC-derived neuronal subtypes showed different ceramide composition. For example, glutaminergic neurons contained higher levels of C_16_ and C_18_ ceramides compared to GABAergic and motor neurons. C_24_ ceramide was shown to be higher in glutaminergic, less abundant in GABAergic, and with the lowest levels in motor neurons (35). The iPSC-derived neuronal and glial cells are a valuable tool; however, the lipidomics data obtained from these cells need to be interpreted with caution and validated in future measurements in primary neuronal and glial cultures. The development of spatial lipidomics techniques for direct measurements of ceramides in future research will provide the ultimate information for the distribution of individual ceramides in neuronal and glial cell populations.

Short-chain ceramides have a length from 2 to 10 carbon atoms, from which exogenous C_2_ and C_6_ ceramides are most studied (Fig. 2) (194, 195). Short-chain ceramides have been described as water-soluble and permeable to the membrane (196, 197), or by Goñi *et al.* (195, 198) as insoluble swelling amphiphiles. This discrepancy could be attributed to the concentrations that were used in those studies (196, 198). One study described a very low amount of C_2_ ceramides in mouse brain (±10 pmol/g) and liver (±25 pmol/g) (199). New research with measurements based on higher sensitivity methods will be needed to confirm this finding. The majority of research with short-chain ceramides uses exogenous synthetic compounds in experiments to mimic the effect of long-chain ceramides (Fig. 2) (200). Interestingly, it has been shown that short-chain ceramides can cross the blood-brain barrier (201). Exogenous C_2_ ceramides can induce autophagy in neuronal cell cultures and contribute to neurotoxicity (183). For example, C_2_ ceramide induces caspase-3 independent cell death and autophagy in SH-SY5Y human neuroblastoma cell line. In primary astrocytes, on the other hand, exogenous C_2_ ceramide inhibits reactive oxygen species production, increases antioxidant enzymes, activating MAPK signals and prevents cell death (202). Similar results were obtained with human astroglioma cells (202, 203). In addition, it has been shown that short-chain ceramides (C_2_, C_6_, and C_8_) are associated with anti-inflammatory responses in the nervous system, while long- and very long-chain ceramides (C_14_ to C_24_) with a proinflammatory role (Fig. 2) (204, 205, 206). One example for the anti-inflammatory responses of exogenous short-chain ceramides is the inhibition of proinflammatory cytokine generation in lipopolysaccharide-stimulated microglial cells (207). Similar to their effect in astrocytes, exogenous C_2_ ceramide suppresses microglial activation, by inhibiting reactive oxygen species, MAPKs, PI3K/Akt, and Jak/STAT pathways (207).

The long-chain ceramides have a length from 12 to 20 carbons, from which C_16_ and C_18_ are the most studied ceramide species (Fig. 2) (208). Long-chain ceramides have been implicated in neuronal apoptosis (209, 210). There are several detailed reviews on the role of ceramide in apoptosis and neurodegenerative disorders (33, 194, 211, 212, 213, 214, 215). Interestingly, ceramides were shown to induce apoptosis or axonal development depending on the developmental stage of the neurons and the expression levels of neurotrophic receptors (211, 216, 217). In the nervous system, C_16_ ceramide has been linked to learning and memory (218). For example, in mice, early life exposure to C_16_ ceramide results in better learning and short-term memory in adulthood (218). In the same study, early life exposure to C_16_ ceramide showed an improvement in learning and memory behaviors in an Alzheimer’s disease mouse model. A study in patients with multiple sclerosis showed an opposite effect for plasma C_16_ ceramide, high levels of which were associated with the disease pathology (219). Moreover, in a mouse model of multiple sclerosis, neuronal deletion of *Cers6* and *Cers5*, which produce C_16_ ceramide, showed milder encephalomyelitis symptoms (220). Deletion of *Cers6* in the neurons decreased neurological deficits and axonal damage (218, 220). In a *postmortem* brain tissue of patients with Parkinson’s disease, in particular in the anterior cingulate cortex, it was found that total ceramide and sphingomyelin were reduced and there was a shift toward ceramides with a shorter acyl chain, shifting from C_24_ and C_24:1_ to C_16_ and C_18_ (221). However, in a different study, in the plasma from patients carrying a mutation in the glucosylceramidase beta gene, which is associated with Gaucher and Parkinson’s diseases, the total levels of ceramide and sphingomyelin were increased (222). In Alzheimer’s disease, it has been shown that in the earlier stages there is accumulation of ceramide, while at the later stages of the disease ceramide levels decreased (42). This was attributed to low levels of ceramides in the middle frontal gyrus white matter of the patients at the later stages of the disease (42). Moreover, accumulation of long-chain ceramides, C_16_ and C_18_, the very long-chain C_24:1_ ceramide (Fig. 2), and cholesterol have been associated with Alzheimer’s disease and aging (210, 223). In those studies, it was shown that ceramide and cholesterol metabolism were affected by membrane-associated oxidative stress due to amyloid-beta deposition, contributing to neurodegenerative pathology. In a different study, cerebrospinal fluid C_18_ ceramide was proposed to be a marker for early stages Alzheimer’s disease and inflammation (224). Similar results were published from patients with multiple sclerosis. In their cerebrospinal fluid and plasma C_16_ and C_24_ ceramides were increased (41). Several studies have addressed the contributions of long-chain ceramides in neuroinflammatory conditions (205, 225). For example, C_16_ ceramide is increased in post-mortem tissue, especially in astrocytes, of patients with frontotemporal dementia, Niemann-Pick’s disease, and Parkinson’s disease with dementia (225). The proinflammatory mechanisms of long-chain ceramides involve stimulation of proinflammatory cytokines and activation of NF-κB signaling, reviewed in (205, 226).

The very long-chain ceramides are defined as containing 22 to 26 carbon atoms (C_22_ to C_26_) (Fig. 2) (227, 228). Very long-chain ceramides have been associated with age-related diseases (208, 229). For example, high levels of C_24_ ceramide have been found in the brain and in the plasma of Alzheimer’s disease and multiple sclerosis patients (208, 229, 230, 231, 232, 233). In contrast, the brain of an adult *Cers2* deficient mouse contains low levels of very long-chain ceramides, which resulted in progressive myelin loss, myelin sheath defects, and cerebellar degeneration (234). Moreover, in an oligodendrocyte specific *Cers2* knock out mouse model, there was a reduction in myelin thickness as well as a reduction in the percentage of myelinated axons (235). The *Cers2* knock out mice showed a reduction in C_22_ and C_24_ myelin sphingolipids, such as galactolipids and sulfatides. Interestingly, the reduction of very long-chain sphingolipids was compensated with an increase in C_18_ sphingolipids. The compensation with C_18_ ceramide was not sufficient to restore the myelin loss highlighting the importance of very long-chain ceramides and very long-chain ceramide moieties in complex sphingolipids for myelin integrity (235). This is not surprising considering that the hydrophobic moieties of galactosylceramides are very long-chain ceramides (187, 234, 236). Interestingly, reduced levels of very long-chain ceramides and sphingomyelins (C_24_ and C_26_) due to reduced levels of CerS2 mRNA, protein, and activity were measured in fibroblasts isolated from patients diagnosed with progressive myoclonic epilepsy (237).

## Atypical sphingolipids in the nervous system

In addition to L-serine, the precursor of canonical sphingolipids, SPT can also utilize L-alanine and glycine as amino acid substrates (26, 238). SPT has higher affinity for its L-serine substrate (K_m_ ∼ 0.40–1.2 mM) (60, 238, 239, 240). L-alanine (K_m_ > 9.6 mM) or glycine are less preferred substrates, which are precursors of atypical sphingolipids, 1-deoxysphingolipids and 1-deoxymethyl sphingolipids, respectively (Fig. 2) (26, 238). As a result of SPT’s lesser affinity to L-alanine and glycine under normal physiological conditions, the generation of 1-deoxysphingolipids and 1-deoxymethyl sphingolipids is lower than the generation of canonical sphingolipids (26, 238). 1-deoxyceramides lack an OH group at the C_1_ position, therefore they cannot be converted to complex sphingolipids, nor be catabolized as the canonical sphingolipids through S1P degradation by S1P lyase (241, 242). 1-Deoxysphingolipids were considered to be dead-end metabolites until it was discovered that they can be catabolized by cytochrome P450 4F enzyme (242). However, the P450 degradation pathway for 1-deoxysphingolipids is slower than S1P lyase degradation of the canonical sphingolipids (242). As a consequence, if produced in excess, the 1-deoxysphingolipids accumulate (26).

1-Deoxysphingolipids and 1-deoxymethyl sphingolipids were originally identified in the context of hereditary sensory neuropathy type 1 (HSAN1) and shown to be neurotoxic when produced in excess (26). HSAN1 is an autosomal dominant, slowly progressive neurological disease. HSAN1 neuropathic symptoms are loss of sensation, including temperature in the extremities, accompanied by shooting pain, skin ulcers, infections, motor neuron and axonal degeneration, followed by atrophy and weakness of the distal muscles (243). HSAN1 is caused by mutations in *SPTLC1* and *SPTLC2*, the majority of which being missense mutations; for example, C133W in *SPTLC1* and S384 and A182P in *SPTLC2* (26, 244, 245, 246, 247). These mutations result in SPT catalytic shift due to increased SPT affinity to the less preferred substrates, L-alanine and glycine, leading to the production of atypical sphingolipids in excess (238, 246, 248).

Increased levels of 1-deoxysphingolipids also have been observed in metabolic disorders and in type 2 diabetic neuropathy (249, 250, 251). Diabetic neuropathy is a sequela in diabetes. In diabetic neuropathy, the sensory, autonomic, and motor axons are affected (252). Clinical pathology shows small fiber nerve damage and symptoms include burning and shooting pain, pinprick sensation, paresthesia, dysesthesia like hyperalgesia and allodynia, and poor balance, which can lead to falls (253, 254). In addition, in diabetic neuropathy, there is large fiber damage that often results in numbness and loss of sensation, resulting in foot ulcers and infections (253, 254). Increased levels of 1-deoxyceramides, 1-deoxysphinganine, and 1-deoxysphingosine have been found in the plasma of diabetic neuropathic patients and in the plasma of a rat model of diabetes (255, 256). The exact mechanism for the increased levels of 1-deoxysphingolipid in diabetic neuropathy is not known. However, supplementation with L-serine, the high affinity SPT substrate, decreased 1-deoxysphingolipid levels in the diabetic rat model (255). In addition, L-serine levels were lower in patients with obesity, and type 1 diabetic neuropathy (251). These findings suggest that increased levels of 1-deoxysphingolipids in diabetic neuropathy are likely due to an L-serine deficiency.

Oral L-serine supplementation has shown positive results not only in mouse diabetic neuropathy models, but also in HSAN1 mouse model and in HSAN1 patients (257, 258). In a HSAN1 mouse model, specifically with the C133W mutation, 10% L-serine enriched-diet reduced 1-deoxysphingolipid levels, improving motor and sensory deficits (257). In HSAN1 patients, L-serine supplementation resulted in decreased levels of 1-deoxysphingolipids like the mouse model (257). These initial results were confirmed in a randomized clinical trial, where L-serine supplementation was tested in HSAN1 patients (258). The results from the clinical trial showed that L-serine supplementation resulted in decreased plasma levels of 1-deoxysphingolipids and, importantly, in improvement of sensory and motor neuropathy measured by the Charcot-Marie-Tooth Neuropathy Score. The clinical study did not identify any serious adverse effects due to L-serine supplementation.

Work from our laboratory identified 1-deoxysphingolipids as intermediates in taxane-induced peripheral neuropathy (259, 260). Taxanes, paclitaxel and docetaxel, are chemotherapeutic agents that can lead to peripheral neuropathy, a major dose-limiting side effect (261). The clinical symptoms of taxane-induced peripheral neuropathy include shooting pain in the extremities, numbness, muscle weakness, loss of sensory perception, and motor deficits (261, 262). In cell cultures and in dorsal root ganglia neurons of mice, taxane treatment, with both paclitaxel and docetaxel, resulted in SPT upregulation and increased 1-deoxysphingolipids production (259, 260). Importantly, in patients, paclitaxel treatment resulted in association of high plasma levels of the very long chain 1-deoxyceramides (C_24_ and C_22:1_) with peripheral neuropathy symptoms (259). Results from the mouse model of taxane-induced neuropathy, showed that 1-deoxysphingolipids were increased in dorsal root ganglia but not in the spinal cord, limiting the effects of taxanes to the peripheral nervous system (260). Of note, taxanes cannot cross the blood brain barrier (263, 264, 265).

Because of their role in peripheral neuropathies, atypical sphingolipids are studied for their neurotoxicity. Although not entirely elucidated, some of the molecular and cellular mechanisms that have been suggested for 1-deoxysphingolipids neurotoxicity are reduced cell viability and proliferation, disrupted calcium handling in the endoplasmic reticulum and mitochondria, mitochondrial dysfunction and loss of membrane potential (241, 266, 267, 268, 269, 270, 271). 1-Deoxysphingolipid treatments in cells were shown to cause cytoskeletal changes, such as loss of actin stress fibers and abnormal actin dynamics, as well as altered neurofilament and microtubule structures (241, 266, 267, 272). 1-deoxysphingolipids were shown to cause damage to cultured motor and sensory neurons manifested by altered cell architecture, reduced neurite length and number, likely due to imbalance between neurite outgrowth and retraction (26, 241, 270, 273). In addition, neurite swellings, and simplified dendritic arborization were observed because of 1-deoxysphingolipid treatments (260, 273).

Very little is known about physiological functions of atypical sphingolipids, such as 1-deoxysphingolipids, in nonpathological conditions. One study, in cardiomyocytes and in lymphatic endothelial cell lines, describes that 1-deoxysphingosine can act as a ligand activating nuclear hormone receptors, nuclear receptor subfamily 2 group F member 1 and 2 (NR2F1 and 2) by increasing their transcription (274). This study implies that 1-deoxysphingolipids physiological functions can relate to the nervous system, as it is known that NR2Fs are involved in regulating oligodendrocyte development, axonal guidance, arborization in the peripheral nervous system and formation of GABAergic interneurons (274).

## Biochemical modifications of the LCBs and ceramides affecting their biophysical properties, function, and pathology in the nervous system

### Hydroxylation

Ceramides can be hydroxylated either on the FA or on the LCB moiety (Fig. 2) (275, 276). The OH group could be at a different position on the FA moiety. However, in eukaryotes, the FA hydroxylation of ceramides occurs primarily at position C_2_ (α-hydroxylation) (277, 278, 279). The reaction is catalyzed by the enzyme fatty acid 2-hydroxylase (FA2H). In addition to the C_2_ position, the FA moiety can be hydroxylated at the terminal carbon, that is, ω-hydroxylation (196).

In mammals, hydroxylation in the LCBs, occurs at the C_4_ position catalyzed by the enzyme delta(4)-desaturase, sphingolipid 2 (DEGS2), that can act as a desaturase and hydroxylase, generating ceramides or phytoceramides, respectively (Fig. 2) (280, 281). The LCB in phytoceramides is phytosphingosine (t18:0), which is prevalent in plants, fungi and yeast, but rarely found in animals (33, 44, 52). Low amounts of phytoceramides were found in the brain (281). Interestingly, a study on metabolomic patterns of different pituitary stalk lesions identified phytosphingosine as one of the metabolites in the cerebrospinal fluid which showed differences between the types of lesions and can potentially be used as a marker to identify them (282).

The hydroxylation level of ceramides has a direct effect on the hydrogen bond interaction of ceramides with other membrane lipids, such as glycerophospholipids and cholesterol and influences membrane properties (283). Studies in skin have shown that ceramide hydroxylation stabilizes its interactions with other membrane lipids influencing membrane properties such as membrane fluidity, permeability, diffusion, and leakage (283, 284). The effects of ceramide hydroxylation depend on the position of the hydroxyl group, whether it is on the FA chain or on the LCB (277). If the additional hydroxyl group is on the LCB moiety, like in phytoceramides, this leads to increased membrane fluidity. However, if the additional hydroxyl group is on the FA chain, membrane fluidity is decreased. It has been shown that an LCB with more than one hydroxyl group can strengthen lipid interactions, resulting in enhanced permeability barrier in epithelial tissues (285). In addition, the hydroxylation of ceramide can alter membrane organization, including microdomain formation, such as lipid rafts, as well as affect membrane protein distribution (283).

Sphingolipids with α-hydroxylated ceramide moiety are abundant in the nervous system, especially in myelin, where they have been found to play a role in maintaining the stability of the myelin sheath (286). FA2H, the enzyme catalyzing the hydroxylation of ceramide FA moiety, is expressed in myelinating cells - oligodendrocytes and Schwann cells (278, 287). Mutations in the *FA2H* gene can lead to partial loss of function, manifested in enzyme deficiency leading to the neurodegenerative disease fatty acid hydroxylase-associated neurodegeneration (FAHN) or hereditary spastic paraplegia 35 (HSP35/SPG35) (288). FAHN is characterized by motor dysfunction such as ataxia, dystonia, spasticity, oculomotor degeneration, as well as seizures and intellectual impairment (288, 289). Due to the α-hydroxylated sphingolipid abundance in myelin, they have been implicated in its long-term maintenance and integrity, and in axonal support (278, 286, 290). Observations based on neuroimaging and MRI data associated deficiency of 2-hydroxylated sphingolipids with abnormal white matter, manifested as white matter lesions, thinning in corpus callosum, cerebellar atrophy, myelin and axonal degeneration (287, 290, 291, 292, 293).

### Saturation

The presence of a double bond in the LCBs changes the structure of ceramides and complex sphingolipids affecting their function. The enzyme dihydroceramide Δ4-desaturase 1 (DEGS1) introduces a trans (E) double bond in the C_4-5_ position of dihydroceramides, generating ceramides (Figs. 1 and 2) (63, 87). This trans (4E) double bond is required for ceramide signaling functions (50). It was shown that dihydroceramides are less potent as signaling molecules inducing apoptosis than ceramides (63, 294, 295). Therefore, the presence of the double bond in the ceramides increases their biological activity compared to dihydroceramides (296). Disruption of DEGS1 activity was shown to lead to pathology (297). For example, low DEGS1 activity leads to an accumulation of dihydrosphingolipids and reduced levels of ceramides, which have been linked to neuronal dysfunction. Mutations in the *DEGS1* gene have been shown to cause hypomyelinating leukodystrophy, a disease characterized by the deficiency in myelin deposition (297, 298, 299, 300). Disruptions in the metabolism of dihydroceramides and ceramides lead to imbalances in their levels, which has been implicated in neurological conditions. For example, high levels of dihydroceramide have been found in cerebrospinal fluid of Alzheimer’s disease patients (297, 301). In Parkinson’s disease, ceramides have been found to be increased in the serum, in the postmortem cerebrospinal fluid and in the primary visual cortex. For further information on ceramide and complex sphingolipids in Parkinson’s disease we recommend the review by Custodia *et al.* (302). A mutation in DEGS1, leading to increased levels of dihydrosphingolipids, was identified as the cause in a patient presented with seizures, microcephaly, progressive neurological dysfunction, neurogenic muscular atrophy, hypomyelination, and thinner white matter (303). These findings corroborate the important role for DEGS1 in the brain, and its role in maintaining dihydroceramide/ceramide homeostasis essential for brain development and function (35, 196). The lack of the 4,5-trans-double bond in dihydroceramides and in the other dihydrosphingolipids reduces membrane fluidity (55, 296). Therefore, the ratio of saturated (dihydroceramides) *versus* unsaturated (ceramides) influences the rigidity of the membranes (277). The dihydroceramides/ceramides ratio varies in different tissues. Higher levels of dihydroceramides support rigid membranes in the skin to maintain its barrier properties, unlike in the brain where unsaturated ceramide species can contribute to more fluid membranes.

Changing the C_4-5_ trans- to a nonnatural cis-double bond configuration in the LCB moieties of ceramides affects their membrane packing behavior modulating the membrane elastic properties and membrane domain formation (277, 295, 304, 305). The natural trans configuration of the C_4-5_ double bond of ceramides LCB moiety has been shown to allow for the formation of more hydrogen bonds with other membrane lipids. This allows for a higher ordered phase and packing of the membrane bilayer, which results in higher membrane resistance to bending (305).

The presence of the double bond in ceramides and its configuration can affect the affinity of enzymes that use ceramides or dihydroceramides as their substrates. It has been shown that most of the ceramidases have higher affinity toward ceramides with a 4,5-trans-double bond and show drastically lower activity toward saturated dihydroceramides (71). In addition, changing the double bond configuration from trans to cis, results in reduced ceramides affinity.

The degree of saturation of the LCB also influences its function. Sphingadienine is an LCB containing two double bonds, one trans (E) at C_4,5_-position similar to sphingosine, and a second distinct, cis (Z), at C_14-15_ position (Fig. 2) (306). The enzyme responsible for introducing the cis-double bond at the position C_14-15_ is the fatty acid desaturase 3 (FADS3) (307). The FADS3 generates sphingadienine containing ceramides from ceramides containing sphingosine, however, there is still a debate in the field if free sphingosine can also be a substrate for FADS3 (285, 308). Furthermore, sphingadienine-containing ceramides are predominantly metabolized to sphingadienine-containing sphingomyelins (285). The second cis-double bond in sphingadiene can weaken lipid–lipid interactions, affecting the composition of lipid raft microdomains (285). An increased level of sphingadienes was found in the hippocampus of sphingosine kinase 2 KO mice (309). Free sphingadienines can have neurotropic and anti-inflammatory effects (55). Although, sphingadienines were identified in 1969 (306), its metabolism was only recently addressed. There are still questions remain regarding the difference between sphingadienine metabolism and the metabolism of other LCBs such as sphingosine (285). The metabolism of sphingadienine is a novel avenue of research that needs to be further explored in the nervous system.

In sphingosine, a canonical LCB, the double bond is trans (E) at C_4,5_-position (Fig. 2) (63). In contrast, it has been found that 1-deoxysphingosine can have a 14Z double bond (307). It is still debated whether 1-deoxysphingosine can also exist as a 4E positional isomer (29). Both 1-deoxysphingosine positional isomers, 4E and 14Z, have the same mass-to-charge ratio (m/z), making it difficult to distinguish by mass spectrometry. Interestingly, 1-deoxymethylsphingosine has a 3E double bond, like the 4E double bond of canonical sphingosine (29). The 14Z position of the double bond in 1-deoxysphingolipids indicates that their metabolism likely differs from the metabolism of the canonical sphingolipids. Until very recently, it was not known which enzyme was responsible for introducing the 14Z double bond in 1-deoxysphingolipids. It was suggested that since sphingadienine also contains a 14Z double bond, it is possible that the same enzyme could be involved in the introduction of the 14Z double bond in 1-deoxysphingolipids. Indeed, by using several diverse methods, including enzymatic assays, metabolic tracing assay and lipidomic profiling combined with genome-wide association study, it was shown that FADS3 is the enzyme responsible for introducing the 14Z double bond in both sphingadienines and 1-deoxysphingosine (14Z) (285, 307).

## Conclusions

In this review, we aimed to emphasize the importance of sphingolipids, and sphingolipid precursors, the LCBs and ceramides, in nervous system homeostasis. The functional significance of sphingolipid precursors’ structural diversity is starting to emerge in recent research (Table 2). Moreover, disturbance of just one metabolic node, which contributes to the generation of LCBs and ceramides or to their structural modification can lead to neurological pathologies. Contributing to these layers of complexity, individual LCBs, ceramides, atypical 1-deoxysphingolipids, and the enzymes that generate them have specific tissue and cell type distribution shaping their membrane properties and modulating the function of their membrane proteins. We chose to highlight the importance of sphingolipid precursor metabolites, LCBs and ceramides, in the nervous system because historically the main body of research on sphingolipids in the nervous system was focused on complex sphingolipids and their related neurological pathologies. The free LCBs and ceramides and the contribution of their structural properties to the overall function of complex sphingolipids in the nervous system is underexplored and requires attention moving forward in future research. Another unresolved question is whether short-chain ceramides naturally occur in the nervous system, especially considering the extensive body of research with exogenous short-chain ceramides and the possible implications for their function in the nervous system.

**Table 2:** Sphingolipid structure-function effects in the nervous system

Structural characteristics		Effects	References
Chain-length of the LCB	C_20_ LCBs	*increased levels lead to*: • neurodegeneration in the brain and retina • abnormal accumulation of neuro filaments in cerebellar white matter • abnormal membrane structures and vacuolization	(169)
	C_18_ LCBs	*elevated levels lead to*: • neurodegeneration • ataxia phenotype • pan-neuronal membrane abnormalities • accumulation of lipofuscin	(181, 182)
Chain-length of ceramide’s fatty acyl moiety	short-chain ceramides (C_2_ to C_10_)	• can cross the blood-brain barrier • exogenous C_2_ ceramides can induce autophagy and contribute to neurotoxicity • associated with anti-inflammatory responses.	(183, 201)
	long-chain ceramides (C_12_ to C_20_)	• the most abundant ceramides in the brain • proinflammatory • proapoptotic • have effect on learning and memory • associated with Alzheimer’s disease and aging.	(204, 205, 206, 209, 210, 218, 223)
	very long-chain ceramides (C_22_ to C_26_)	• associated with age-related diseases • affect myelin integrity *low levels result in:* • progressive myelin loss • myelin sheath defects • cerebellar degeneration	(208, 229, 234)
Hydroxylation of the LCB	lack of hydroxyl group at position C_1_ (atypical 1-deoxysphingolipids)	*production in excess results in*: • Hereditary Sensory and Autonomic Neuropathy Type 1 • diabetic neuropathy • taxane-induced peripheral neuropathy	(26, 249, 250, 251, 259, 260)
	Hydroxylation at C_4_ (phytosphingosine, phytoceramide)	• low levels in the brain • identified in pituitary stalk lesions • increase membrane fluidity	(281, 282)
Hydroxylation of ceramide’s fatty acyl moiety	C_2_ hydroxylation (α-hydroxylated sphingolipids)	• Effect on membrane fluidity • abundant in myelin • role in myelin stability, long-term maintenance, and integrity • axonal support *deficiency associated with*: • abnormal white matter and lesions • thinning in corpus callosum • cerebellar atrophy • axonal degeneration	(277, 278, 286, 287, 290, 291, 292, 293)
Saturation of the LCB	C_4-5_ trans double bond	• decreases membrane fluidity • promotes domain formation • affects enzyme affinity • required for ceramide signaling functions and biological activity *lack of the C*_*4-5*_ *trans double bond (dihydroceramides), dihydroceramides increased levels result in:* • hypomyelination, hypomyelinating leukodystrophy • seizures • microcephaly • progressive neurological dysfunction • neurogenic muscular atrophy • thinner white matter • associated with Alzheimer’s disease	(50, 55, 63, 87, 296, 297, 298, 299, 300, 301, 303)
	C_14-15_ cis double bond	• found in atypical 1-deoxysphingolipids • possible role in neurotoxicity	(307)
	two double bonds, trans C_4-5_ and cis C_14-15_ (sphingadienine)	• neurotropic • anti-inflammatory • effect on lipid–lipid interactions and lipid raft microdomains	(55, 285)

An interesting aspect of LCB and ceramide metabolism is the ability to utilize compensatory mechanisms to preserve their homeostasis. These compensatory mechanisms are particularly important in neuronal development and in neurodegeneration. Of note is that these compensatory mechanisms often change the balance of the individual LCB or ceramide species, which, while keeping the cells of the nervous system alive, often do not fully support their function and compromise overall nervous system homeostasis. A deeper understanding of these mechanisms will enable the development of future therapeutic strategies aiming at fine tuning sphingolipid enzyme activities to maintain sphingolipid and neuronal homeostasis.

In the last decade, new research revealed that SPT has amino acid substrate promiscuity leading to the production of neurotoxic atypical sphingolipids, which accumulate in pathological conditions. Understanding the metabolism of those atypical sphingolipids provides a mechanistic foundation to address important clinical pathologies such as peripheral neuropathies. 1-Deoxysphingolipids serve as a good example of the importance of studying sphingolipids and their role in physiology, given the fact that up until today, little is known about the function of 1-deoxysphingolipids in nonpathological situations. In addition, the regulation of SPT by ORMDLs is an example of how cells maintain ceramide and sphingolipid homeostasis by sensing elevation of sphingolipid metabolites (61, 62). Research on ORMDLs is an exciting novel direction in the sphingolipid field. It is not clear if ORMDLs can sense only ceramide accumulation or whether there are other metabolites, such as complex or atypical sphingolipids, that might play a part as well.

An example of the importance of the LCB structure for its function is the chain length of the LCBs. The observation that C_20_ LCBs accumulate throughout life in the brain but decrease in plasma suggests that they are plasma markers with inverse correlation for brain aging. This is particularly interesting considering current paucity of plasma neurological markers related to brain aging.

The biochemical modifications of LCBs and ceramides have significant effects on their function and that of their sphingolipid derivatives; however, the specific effects of these modifications and their implications on sphingolipids within the nervous system remain still understudied. Addressing that and other outstanding questions in future research on sphingolipid precursors could be advanced by emerging new tools and techniques. In the last couple of decades, there was a significant progress in advancing mass spectrometry measurements of individual LCBs, ceramides, and atypical sphingolipids. The arsenal of mass spectrometry techniques includes high quality quantitative targeted and direct infusion, so called “shotgun”, lipidomics techniques. In addition, cross-link ready sphingolipid probes are becoming available to address the lipid–lipid and lipid–protein interactions in combination with proteomics and imaging techniques (310, 311, 312, 313). These novel techniques will boost research to further understand the effects of biochemical modifications and sphingolipid profiling of LCBs and ceramides in the nervous system. It will further characterize their cellular, molecular, and physiological functions, and their role in neurological diseases.

## Conflict of interest

The authors declare that they have no conflicts of interest with the contents of this article.
